# On standardization of controls in lifespan studies

**DOI:** 10.18632/aging.205604

**Published:** 2024-02-27

**Authors:** Olga Spiridonova, Dmitrii Kriukov, Nikolai Nemirovich-Danchenko, Leonid Peshkin

**Affiliations:** 1 Department of Systems Biology, Harvard Medical School, Boston, MA 02115, USA

**Keywords:** animal disease models, survival modeling, aging, data standardization

## Abstract

The search for interventions to slow down and even reverse aging is a burgeoning field. The literature cites hundreds of supposedly beneficial pharmacological and genetic interventions in model organisms: mice, rats, flies and worms, where research into physiology is routinely accompanied by lifespan data. However, when experimental animals from one article live as long as controls from another article, comparing the results of interventions across studies can yield misleading outcomes. Theoretically, all lifespan data are ripe for re-analysis: we could contrast the molecular targets and pathways across studies and help focus the further search for interventions. Alas, the results of most longevity studies are difficult to compare. This is in part because there are no clear, universally accepted standards for conducting such experiments or even for reporting such data. The situation is worsened by the fact that the authors often do not describe experimental conditions completely. As a result, works on longevity make up a set of precedents, each of which might be interesting in its own right, yet incoherent and incomparable at least for the reason that in a general context, it may indicate, for example, not prolonging the life of an average organism, but compensating for any genetic abnormalities of a particular sample or inappropriate living conditions. Here we point out specific issues and propose solutions for quality control by checking both inter- and intra-study consistency of lifespan data.

## INTRODUCTION

In the fascinating realm of aging biology, a critical challenge emerges as we delve into the heart of various studies. The enigma lies in the stark inconsistency that surfaces when we meticulously gather control data across these studies – data from those untouched by interventions, a true baseline to measure against. This inter-study inconsistency becomes evident if we compile the control data (i.e., animals that have not been subject to interventions) across studies. We focus on male C57BL/6J mice - the most widely used inbred strain in biology of aging. According to JAX^®^ Mice and Services/Mouse Phenome Database [1], the median lifespan of C57BL/6J males is 894–901 days, another source [2] indicates a life expectancy of 878 ± 10 days. Our own literature review (Supplementary Figure 1 and Supplementary Table 1) suggests that in various studies the median control lifespan of C57BL/6J animals varies from 600 to 980 days. It is impossible to reliably determine the causes of lifespan variability as it is affected by diet, cage density, temperature, light cycle, etc. In addition, the C57BL/6J line has a long breeding history, and mice taken from different institutions have genetic and physiological features that affect the results of different tests [3]. The road to standardization and quality control of mouse control survival data must necessarily be traversed by someone. In this short article, we offer to go through it with us.

## RESULTS

**Figure 1:**
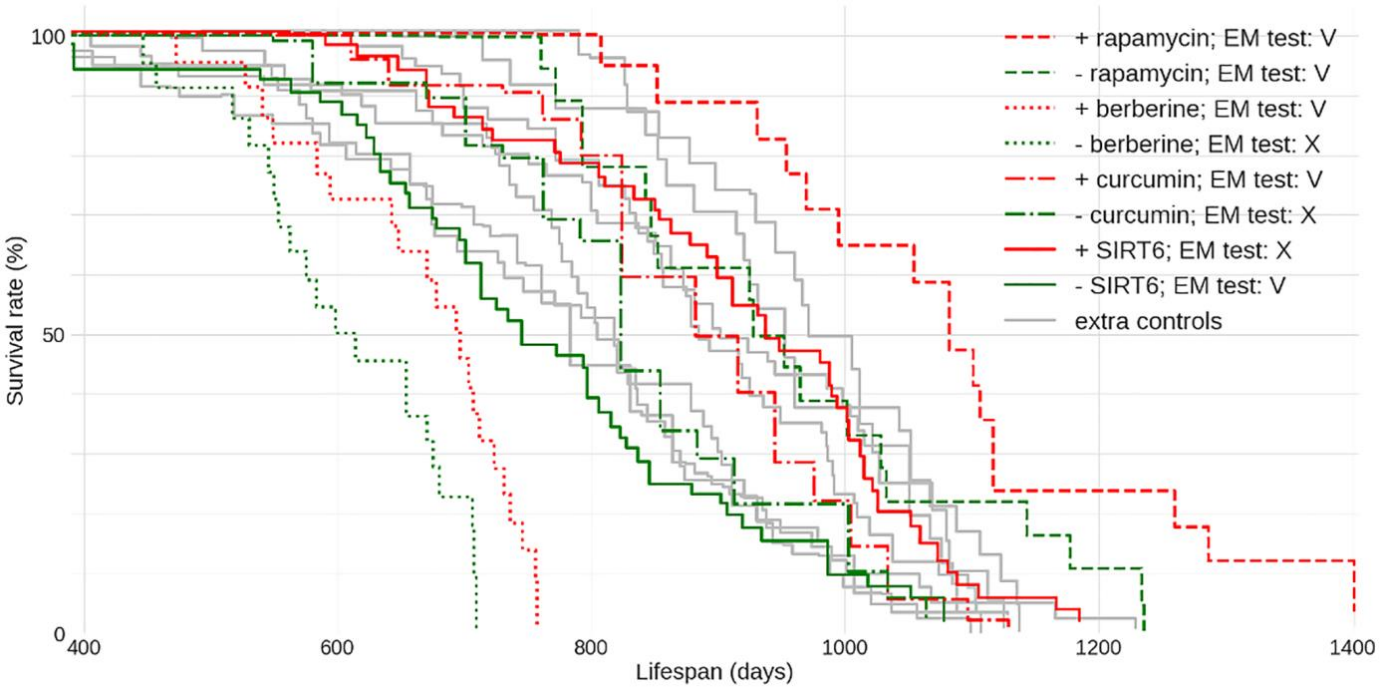
**Lifespan data for C57BL/6J male mice from multiple lifespan extension and other unrelated studies.** Four sample “successful” lifespan extension studies are shown for comparison: the control data is in green and respective intervention data is depicted via the matching stroke (solid or broken) in red. Each colored curve is labeled with its plausibility score *P*. The PMIDs of sources used here are: 27549339 (rapamycin), 31773901 berberine, 17516143 curcumin and 34050173 SIRT6. ‘Meta-control’ data given in gray here are replotted and annotated in Supplementary Figure 1. EM - Extra Mortality test (see Supplementary Materials and Methods) was applied to datasets: V - passed, X - failed with the level of significance α = 0.01.

To further study the life expectancy standard for male C57BL/6J we selected ‘meta-controls’ - control data from several papers where C57BL/6J mice came from two sources: The National Institute of Aging [4–6] and Jackson Laboratories [7, 8] and the origins and conditions of the control animals were described in sufficient detail. Across these studies, the median longevity varies between 800 and 970 days – less than in studies with C57BL/6J males in general. Crucially, even this range far exceeds the typical difference between the median lifespan of a “successful” intervention and control groups, which generally does not exceed 15%. Indeed, when we looked at some of the best-known studies on experimental life extension in C57BL/6J males - we found that in most cases the median lifespan of the experiment, although significantly longer than that of respective control - still did not exceed the longest median lifespan across the ‘meta-controls’ [7] (Figure 1) - one notable exception being the rapamycin [9]. Each dataset also was tested with our newly developed extra-mortality test (see Supplementary Materials and Methods) showing if the given dataset contains implausible instantaneous increase in mortality. A sudden burst of deaths suggests latent issues.

While compiling a comparison like that should be easy, we had to collect these data dealing with entirely different representations each time. It is hard to believe, yet there are no requirements nor data format standards today for submitting the lifespan data along with a publication. We believe that the scientific community needs to take action - to improve the quality of work and the reproducibility of scientific results within aging biology. To this end, we developed the lifespan data and meta-data standard (see Supplementary Materials and Methods). The format is simple yet flexible enough to allow comprehensive data description and re-analysis, we provide encoding for the berberine data from Figure 1 (see Supplementary Materials and Methods). Additionally, we have created a dedicated Web resource “ALEC” (Animal Lifespan Expectancy Comparisons) for accumulation and interactive browsing of lifespan data. We plan to lobby the journals in the field and encourage the authors of previously published and forthcoming publications to adopt this format and to share the data using a central repository. Numerous large datasets of lifespan from studies in mice have already been loaded and are available for interactive browsing and comparison to user-supplied lifespan data. This resource facilitates evaluation of new lifespan data via an instant validation in the context of present knowledge and testing the data with quality control techniques (see Supplementary Materials and Methods) specifically developed and implemented to ALEC. Unfortunately, our attempts to validate the lifespan improvements with other strains did not yield any qualitatively different results from the one we focus on in this study (data not shown).

### Intra-study extra-mortality test

**Figure 2:**
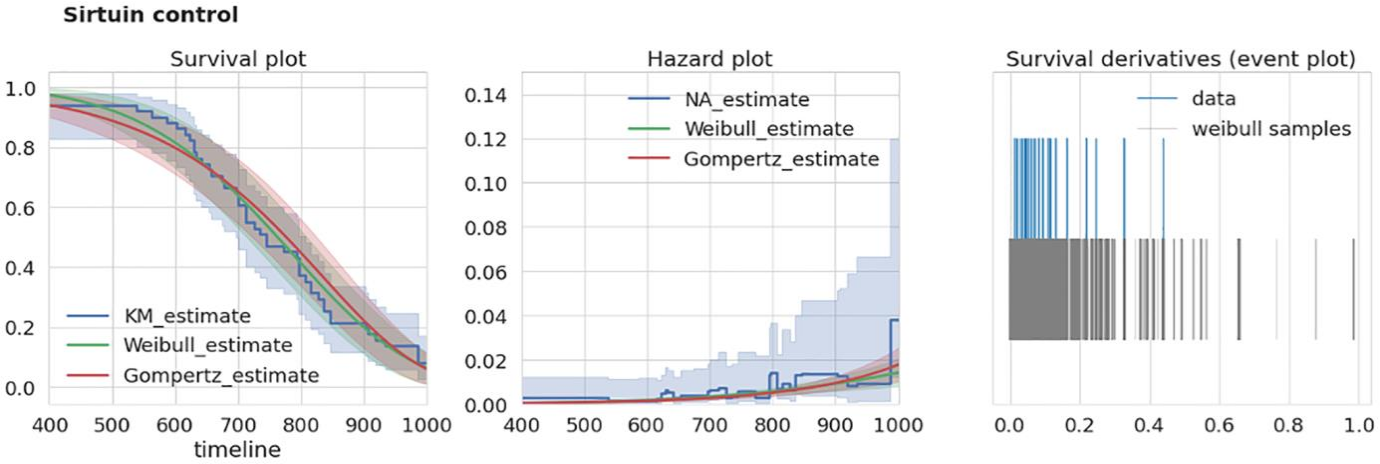
**Example of dataset without over-mortality event.** “Sirtuin control” dataset characteristics. Left, survival curves estimates from nonparametric and parametric estimators. Middle, corresponding hazard (mortality) estimates. Right, event plot (events distribution) of original data and 1000 samples of survival curve derivatives. Abbreviations: KM: Kaplan-Meier estimator; NA: Nelson-Aalen estimator.

**Figure 3:**
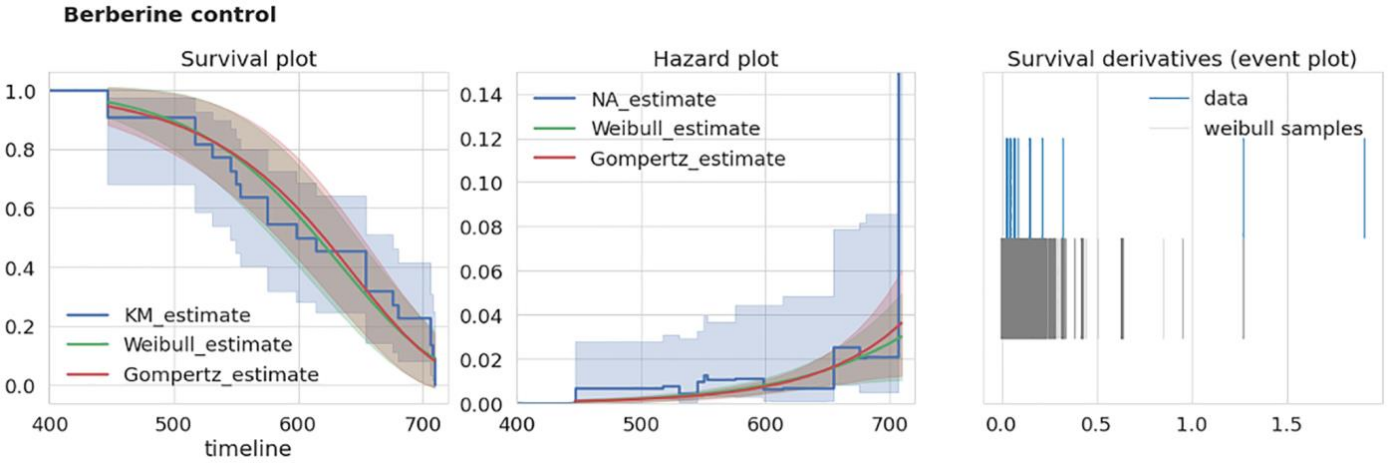
**Example of dataset with over-mortality event.** “Berberine control” dataset characteristics. Left, survival curves estimates from nonparametric and parametric estimators. Middle, corresponding hazard (mortality) estimates. Right, event plot (events distribution) of original data and 1000 samples of survival curve derivatives. Abbreviations: KM: Kaplan-Meier estimator, NA: Nelson-Aalen estimator.

To test the consistency of a given dataset we developed an extra-mortality test checking if the given dataset has an “unexpected” increase in mortality by comparison with two reasonable models of survival curves. To test this for a given dataset we first estimate a time derivative *dŜ/dt* of its corresponding survival function estimate *Ŝ* (we used Kaplan-Meier estimator of *S*). Next, we estimate this derivative using samples drawn from corresponding Weibull and Gompertz approximations of *S*. The example of *S* estimates for two different datasets are given in the left panels of Figures 2, 3. To account for different sample times (i.e., times when researchers recorded the number of dead mice) for Weibull and Gompertz estimates, we used measurement times as in the original experiment, thus binning the observations similarly. By repeating sampling from model distributions 1000 times we compared maximal derivative (vertical step of survival curve) of Kaplan-Meier estimate *dŜ/dt* of original survival curve with model-sampled (Figures 2, 3, right panels). If the maximal derivative in the original study is greater than one from 1000 model samples we consider such a dataset having the extra-mortality artifact. For example, “Sirtuin control” dataset doesn’t have extra-mortality by comparing with 1000 samples from the Weibull model of the dataset (Figure 2, right event plot), and, thus, can be considered as of high quality. In contrast, “Berberine control” has such an artifact (Figure 3, right event plot) and should be analyzed with caution.

To prevent extra-mortality events, we mainly recommend that researchers reduce the time between measurements as much as possible (every day in ideal case). Also, increasing the sample size and maintaining the correct conditions for keeping mice (which is out of scope of the paper) can help avoid abnormal mortality increase.

### Balanced meta-control model for inter-study testing of a treatment effect

Balanced meta-control is a hypothetical control model that harmonizes all accepted meta-controls in ALEC within given strain of across strains. For constructing balanced meta-control we first assume that each control dataset can be approximated with Weibull distribution of parameters *λ* and *ρ* which are scale and shape parameters correspondingly. For fitting the Weibull distribution we use WeibullFitter class of lifelines package [10]. WeibullFitter returns each of the parameters with corresponding standard error of estimate which can be used as intra-study variance for the parameter depending on a sample size and structure of a meta-control dataset. By fitting all meta-controls we obtain a set of parameters *λ* and *ρ* with corresponding standard errors of estimates. For obtaining a balanced average of these parameters we use a meta-regression approach from PyMARE package (meta_regression function) for combining separate estimates with their standard errors. This approach returns balanced average parameters *λ* and *ρ* by accounting variances of Weibull estimates. The resulting parameters can be used for constructing meta-Weibull distribution which is the balanced meta-control (e.g., black curve in Supplementary Figure 1). Next this balanced meta-control model can be used for computing the effect of a new treatment by, for example, comparison of median lifespans of a new survival dataset of treated mice with balanced meta-control median lifespan.

### Inter-study plausibility test for a new control dataset

For testing the plausibility of a new control dataset we propose a procedure relying on the comparison of a new dataset with selected high-quality control datasets. We first assume that each high-quality control dataset can be approximated with Weibull distribution of parameters *λ* and *ρ* which are scale and shape correspondingly. By fitting Weibull to all controls one-by-one we obtain a set of parameters *λ* and *ρ* with corresponding standard errors of estimates.

We next assume that these parameters are drawn from a two-dimensional Gaussian distribution with unknown covariance matrix which we call reference distribution *P*(*λ*, *ρ*). Using the gathered estimates of parameters and their intra-study variances we estimate the covariance matrix and mean vector of the reference distribution. For that we calculate weighted mean, weighted marginal variances and weighted covariance of parameters using their inverse squared standard errors as weights and, thus, accounting differences in inter-study heterogeneity.

**Figure 4:**
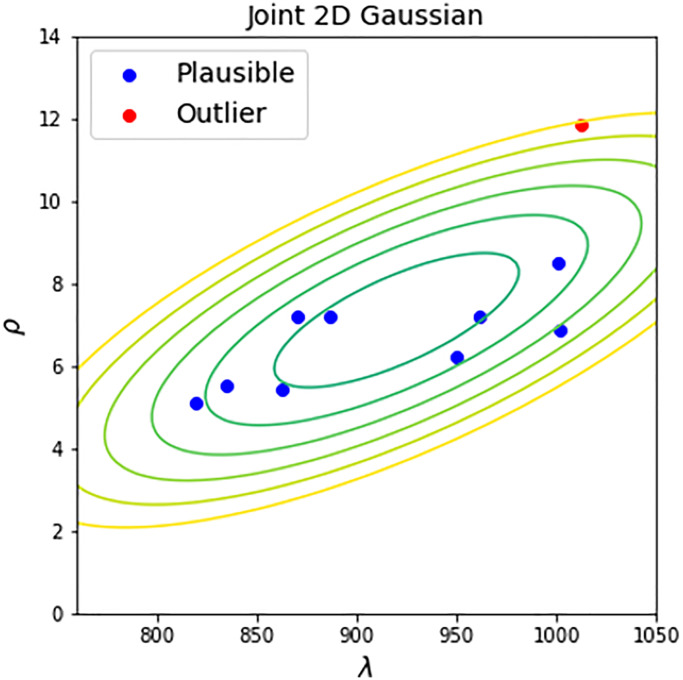
**Inter-study plausibility test.** 2D Gaussian contour plot represents the reference distribution of parameters of high-quality control datasets. The new datasets undergo plausibility Hotelling’s T-squared test with the level of significance equal to 0.01. If a dataset has a Hotelling’s *p*-value larger than 0.01, it is recognized as plausible (blue points). Otherwise, the dataset is recognized as outlier (red points).

Once the reference distribution is computed we may use it for testing the “plausibility” of a new dataset. This can be achieved by estimating its parameters *λ* and *ρ* of corresponding Weibull fit and by computing the distance of the obtained vector of parameters to the corresponding mean vector of the reference distribution by simply applying Hotelling’s T-squared test (Figure 4). The result of the test is a value of statistics (squared Mahalanobis distance) and *p*-value. By accepting a reasonable value of significance α (we propose to use 0.01), this *p*-value can be used for testing the plausibility of a new dataset. If *p*-value is small - the dataset is implausible because of its dissimilarity with high-quality controls.

## DISCUSSION

In summary - in many studies reporting the lifespan extension of C57BL/6J mice, the lifespan of the intervention group appears significantly higher in comparison to the controls - yet is inferior to the lifespan of the control animals of the same strain reported elsewhere. To appreciate the significance of this point, we must consider that the primary practical motivation for experiments in biology of aging is to develop methods for extending the healthy lifespan. Naturally, such methods may differ from those needed for compensatory lifespan extension that is reduced because of genetic abnormalities, environmental challenges or suboptimal living conditions. Of note, the many interventions re-tested in C57BL/6J mice against deep phenotypes of health did not “slow aging” for most parameters monitored [11]. Thus, we posit that the majority of results in biology of aging may be irrelevant to the fundamental aim of this field and must be acknowledged appropriately.

### Code availability

All the analytical instruments utilized in the paper can be found here: https://github.com/shappiron/ALEC_stats. A repository containing the backend and frontend parts of the web-platform is located on https://github.com/imhelle/alec.

## Supplementary Materials

Supplementary Materials and Methods

Supplementary Figure 1

Supplementary Table 1
