# Supplementary material for: On standardization of controls in lifespan studies
Source: Aging (Albany NY). 2024 Feb 27;16(4):3047–55. doi: 10.18632/aging.205604 (PMC10929834; doi:10.18632/aging.205604)
Supplement: Supplementary Materials and Methods [file aging-16-205604-s001.pdf]

SUPPLEMENTARY MATERIALS AND METHODS

Lifespan meta-data format

The proposed data format is structured at three levels of hierarchy: a study meta-data, an experiment meta-data and a raw lifespan data. A given dataset is expected to only contain one study description, defining parameters specific to this study and common to all the experiments in a study (e.g. animal species used), where to find more information about the study (e.g., publication details and identity of whoever submitted the data). If a given dataset federates multiple studies, we expect the data to be broken up into multiple disjoint sets.

Each study is expected to contain several experiments, typically, at least a single intervention and a coupled control experiment but usually more, even though it is not impossible to imagine a single experiment aimed at collecting statistics over normal lifespans. The experiment-specific meta-data defines what distinguishes a given experiment within this study from the others (e.g., specific drug used, gene knock out) as well as auxiliary data description.

Each raw data are trivially a set of numbers, one per line, each defining lifespan of a single individual. This minimalistic design allows for unambiguous data re-analysis. There are cases when additional per-individual data are desired, such as the health state of an animal, whether it was introduced separately or removed early, co-location, accidental injury. These can be provided in a separate CSV file ordered in the same way as the lifespan data, where specifics of the columns/fields are defined in the respective experiment meta-data “remarks” field.

We supply a set of Excel files to provide a template (three files: study, experiment, lifespan) for publishing new data and to illustrate (five files: study.xlsx, experiment1.xlsx, experiment2.xlsx, lifespan1.xlsx, lifespan2.xlsx) the format using one of the studies from Figure 1.
