# Supplementary material for: On standardization of controls in lifespan studies
Source: Aging (Albany NY). 2024 Feb 27;16(4):3047–55. doi: 10.18632/aging.205604 (PMC10929834; doi:10.18632/aging.205604)
Supplement: Supplementary Figure 1 [file aging-16-205604-s002.pdf]

SUPPLEMENTARY FIGURE

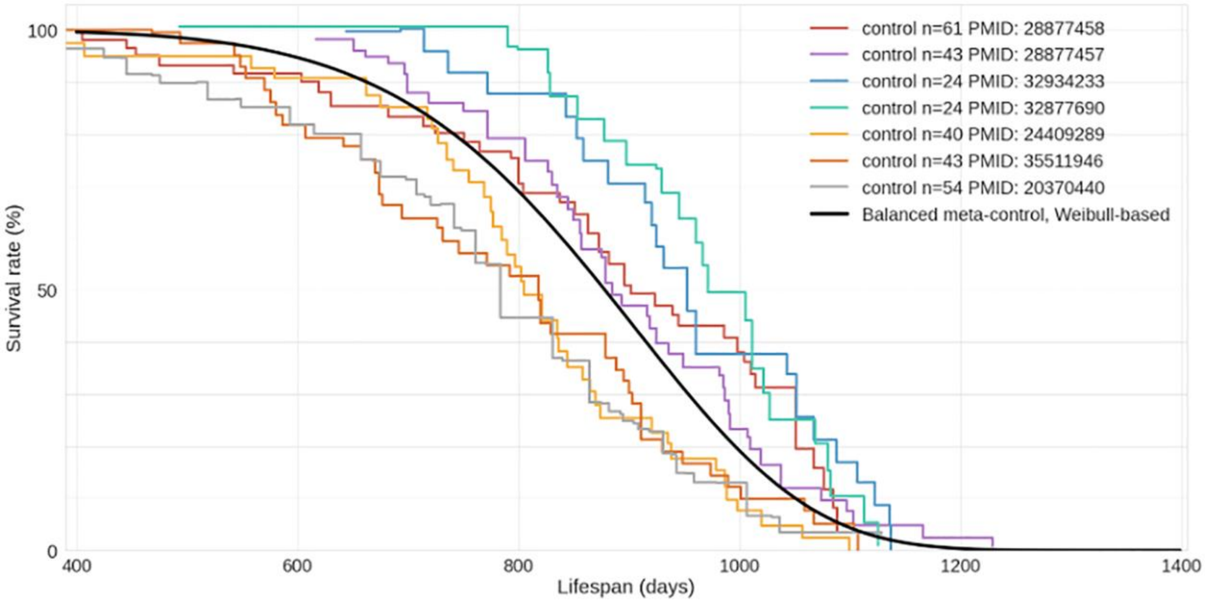

Supplementary Figure 1. Selected meta-controls which were used as the background plotted in gray in the main text Figure 1 with respective source papers cited by PubMed IDs. Black solid line corresponds to a balanced meta-control constructed from parameters of fitted Weibull models of meta-controls (see Supplementary Materials).
