# Supplementary material for: On standardization of controls in lifespan studies
Source: Aging (Albany NY). 2024 Feb 27;16(4):3047–55. doi: 10.18632/aging.205604 (PMC10929834; doi:10.18632/aging.205604)
Supplement: Supplementary Table 1 [file aging-16-205604-s003.pdf]

SUPPLEMENTARY TABLE

Supplementary Table 1. Characteristics of datasets from figure 1 of the main text.

	n	Mean surv time	5% Surv time	Median surv time	95% Surv time
−curcumin	50	830	1033	823	580
+curcumin	50	882	1033	882	640
−berberine	22	605	709	598	446
+berberine	22	661	755	693	527
−SIRT6	52	753	1051	745	392
+SIRT6	51	911	1105	937	647
−rapamycin	18	938	1234	927	679
+rapamycin	17	1087	1401	1082	807
PMID:28877458	61	879	1088	901	474
PMID:24409289	40	815	1020	804	558
PMID:28877457	43	903	1103	884	696
PMID:32877690	24	969	1112	971	826
PMID:32934233	24	944	1135	952	714
PMID:20370440	54	769	1006	783	445
PMID:35511946	43	795	1067	818	548
